# Supplementary material for: How did the characteristics of the growing season change during the past 100 years at a steep river basin in Japan?
Source: PLoS One. 2021 Jul 30;16(7):e0255078. doi: 10.1371/journal.pone.0255078 (PMC8324334; doi:10.1371/journal.pone.0255078)
Supplement: Attachment — Submitted filename: Phenology.docx [file pone.0255078.s001.docx]

This study addresses change of plant phenological events due to climate change. Authors emphasize that the effects of climate change on the phenological events may be greater in a steep river basin than at the scales of the nation and continent.
 They calculated the start (SGS) and end (EGS) dates of the growing season and thus compared the dates between a steep river basin (1420 m a.s.l.) and meteorological station (560 m a.s.l.). Furthermore, they also obtained the green-red vegetation index from satellite image and thus compared the index between both sites.

But there are some questions. First of all, there is a question about Table 3's data. The difference in altitude between the two sites is 860 m. Then the temperature difference according to the altitude should be about 5°C, but there is not that much difference.

Urbanization is known to have a greater impact than a rise in carbon dioxide as a cause of climate change. However, the results of this study indicate that changes in phenological events are occurring faster in site located on the higher altitudes than in the site of lower elevation with high land-use intensity. The authors estimated the background as follows: This possibility is due to the effect of temperature on plant phenology and the difference between vertical and horizontal gradients in temperature sensitivities. But there is no detailed evidence. An explanation of this part should be at the heart of this paper.

It is very difficult to read the data. If the date calculated by the measured temperature and the date obtained by analyzing the satellite image data are tabulated, it will be easier to read the data.

In addition, it would also be meaningful to obtain changes in the spatial distribution of temperature from satellite imaging data and compare them with the results of the GVI.

Finally, the paper could benefit from some editing for grammar and usage in some places—syntax is at times awkward.
