# Supplementary material for: How did the characteristics of the growing season change during the past 100 years at a steep river basin in Japan?
Source: PLoS One. 2021 Jul 30;16(7):e0255078. doi: 10.1371/journal.pone.0255078 (PMC8324334; doi:10.1371/journal.pone.0255078)
Supplement: Attachment — Submitted filename: PONE-D-20-20028R1_Comments.docx [file pone.0255078.s002.docx]

The manuscript entitled ‘How did the characteristics of the growing season change during the past 100 years at a steep river basin in Japan?’ by Nagai et al deals with the growing season in a mountainous region of Japan. Paper is scientific but needs improvement. My observations on the follow up correspondence are as follows:

**Comments**

1. Does number of sunny days play any role in the higher elevation to the phenology? I do agree with the author about the urban heat island explanation but want to see if temperature gradient with available sunny days have any impact needs to be studied.
2. Use of the punctuation marks and conjunction e.g. ‘and’ in the abstract and introduction section need revision [e.g. L-27, 68-69] due to unnecessary use at times.
3. The North arrow is an important component of the map and therefore it must be incorporated in each map.
4. What are the limitations of the study? Elaboration a little on this aspect of the study.
5. L- 210 should be “We used RGB composite images and calculated the green-red vegetation index (GRVI; [54]).”
6. Mention in the article, if any cloud removal method used in the research study to get accurate information from the SENTINEL data.
7. Used acronyms need to be in their full form the first time they are used. This issue is consistent in the whole manuscript.
8. The method used to calculate the trend, correlation result, and significance of the results need to be elaborated. The level of significance used to consider the result ‘significant’ should also be mentioned.
9. Camera images and satellite observation were used to validate the obtained results. Therefore, it is better to make one common section 3.4 for validation through camera image and satellite observation. Otherwise, section 3.4 can be split into two sub-sections i.e. one for camera images and the other one for satellite observation.
10. The conclusions section is very week. It should provide a summary of the whole study, including major results of the study, method, issue addressed.
